# Computational analysis of biological functions and pathways collectively targeted by co-expressed microRNAs in cancer

**DOI:** 10.1186/1471-2105-8-S7-S16

**Published:** 2007-11-01

**Authors:** Yuriy Gusev, Thomas D Schmittgen, Megan Lerner, Russell Postier, Daniel Brackett

**Affiliations:** 1 Department of Surgery, University of Oklahoma Health Sciences Center, Oklahoma City, Oklahoma, USA; 2 Division of Pharmaceutics, Ohio State University, Columbus, Ohio, USA; 3 Veterans Affairs Medical Hospital, Oklahoma City, Oklahoma, USA

## Abstract

**Background:**

Multiple recent studies have found aberrant expression profiles of microRNAome in human cancers. While several target genes have been experimentally identified for some microRNAs in various tumors, the global pattern of cellular functions and pathways affected by co-expressed microRNAs in cancer remains elusive. The goal of this study was to develop a computational approach to global analysis of the major biological processes and signaling pathways that are most likely to be affected collectively by co-expressed microRNAs in cancer cells.

**Results:**

We report results of computational analysis of five datasets of aberrantly expressed microRNAs in five human cancers published by the authors (pancreatic cancer) and others (breast cancer, colon cancer, lung cancer and lymphoma). Using the combinatorial target prediction algorithm miRgate and a two-step data reduction procedure we have determined Gene Ontology categories as well as biological functions, disease categories, toxicological categories and signaling pathways that are: targeted by multiple microRNAs; statistically significantly enriched with target genes; and known to be affected in specific cancers.

**Conclusion:**

Our global analysis of predicted miRNA targets suggests that co-expressed miRNAs collectively provide systemic compensatory response to the abnormal phenotypic changes in cancer cells by targeting a broad range of functional categories and signaling pathways known to be affected in a particular cancer. Such systems biology based approach provides new avenues for biological interpretation of miRNA profiling data and generation of experimentally testable hypotheses regarding collective regulatory functions of miRNA in cancer.

## Background

microRNAs (miRNAs) are single-stranded, non-coding RNAs approximately 22 nucleotides in length that post-transcriptionally regulate gene expression in a sequence specific manner by binding with imperfect complimentarity at multiple sites of 3'-UTR regions of mRNA, thereby facilitating mRNA degradation or inhibiting translation initiation (reviewed in [1,2]). First discovered in C. elegans by Victor Ambros [3], miRNAs are shown to be important negative feedback regulators of many biological processes such as development, differentiation, and proliferation in vertebrates and plants. Aberrant expression of miRNAs in human cancer was first reported in 2002 for leukemia [4].

Since then multiple studies have found aberrant expression profiles of miRNAome in all major human cancers (reviewed in [5]). While several target genes were experimentally identified for some miRNAs in various tumors [6], the global pattern of cellular functions and pathways that are affected by miRNAs in cancer remains elusive.

In our recent studies we analyzed expression profiles of more than 200 miRNAs in human cancer cells lines [7] and samples of pancreatic tumors [8]. Using high-throughput real-time PCR we found over one-hundred miRNAs to be differentially expressed in pancreatic cancer in comparison to normal pancreatic tissue. A cluster of forty-seven most significantly over-expressed miRNAs from this study was included in the current analysis along with 4 other datasets published by others (breast, colon and lung cancers [9], and lymphomas [10,11]).

miRNA expression signatures are shown to be specific and allow classification of tumor type as well as different stages in tumor progression and in some cases predict outcome of a disease [12]. A number of studies have shown that expression of some of the proteins affected in cancer was negatively correlated with the expression of specific miRNAs. Based on these findings, several groups have hypothesized that miRNAs may play important role in tumorigenesis and tumor progression and could function as oncogenes or tumor suppressor genes [4,9]. However, such global interpretation of miRNA expression profiling data is impaired by the lack of high throughput target validation methods and mostly relies upon computational analysis of potential mRNA targets.

Computational algorithms have played a central role in discovery of the majority of miRNAs known to date, as well as in prediction of their targets (reviewed in [13-15]). However, virtually all existing programs generate relatively high levels of false positive predictions (up to a twenty percent). Experimental evidence also suggests that these programs generate some false negative predictions. While bioinformatics methods continue to improve specificity and sensitivity of target predictions, the unresolved challenge still remains to utilize even the most accurate predictions for biologically meaningful interpretation of miRNA profiling data. This problem is due to the fact that the majority of known miRNAs are predicted to target a very large number of transcripts. Each miRNA might have up to several hundred targets. In addition, many transcripts from protein-coding genes are targeted by more than one miRNA and some transcripts might have over a hundred target sites for different miRNAs. It has been estimated that nearly 50% of all human gene transcripts are regulated by relatively small number of 474 miRNAs that are known to date with average of ~200 targets per miRNA [13,14].

This conundrum of target multiplicity has become especially evident with the discovery of a large number of differentially expressed miRNAs in many human cancers [4,9]. A significant number of miRNAs in a range from ten to one-hundred were found to be aberrantly expressed in breast cancer, colon cancer, lung cancer, and other major cancers with a predicted total number of targets ranging from several hundred to as many as several thousand. This again presents a problem for global analysis and the biological interpretation of the regulatory impact of miRNAs in cancer cells. There is a clear need for data reduction methods which would allow reducing the list of targets and determining cellular processes that are most significantly affected by miRNAs in cancer.

The Gene Ontology (GO) enrichment analysis is one of the data reduction techniques that could be used to reduce the number of targets of a large group of co-expressed miRNAs and to find biological functions potentially affected by multiple miRNAs.

The concept of combinatorial target regulation by miRNAs has been discussed in the literature [2,13,14] and was incorporated into several current prediction algorithms such as PicTar [13,14] and miRgate (Actigenics/Cepheid) [16,17]. It is based on experimental evidence that some co-expressed miRNAs may all target the same genes or genes from the same functional categories. Several studies have reported results of computational analysis of functional annotation of genes targeted by single miRNAs [18], all known miRNAs [19,20], or small groups of miRNAs that were selected based on high similarity of "seed" sequences in the 5' region and/or large overlap of predicted target sets [21]. However, in case of experimentally obtained miRNA profiling data these approaches are not very practical when the task is to determine common biological functions and regulatory pathways that are targeted by experimentally detected groups of co-expressed miRNAs. Specifically in cancer such groups of miRNAs are often found to have fewer common targeted genes and not to share similar "seed" sequences.

In this study we address this problem of biological interpretation of miRNA profiling data using systems biology analysis of major biological proccesses, disease categories and signalling pathways that are targeted collectively by co-expressed miRNAs in cancer cells. We assumed that filtering GO categories on the total number of hits by miRNAs targeting the same category would reduce the number of false positive target predictions and at the same time would allow narrowing down the large target lists and determining those biological functions and pathways that are most likely to be affected by co-expressed miRNAs.

## Results

### Data sets

Five groups of co-expressed miRNAs were selected from the literature for this study: 3 groups that were reported by Calin et al. [9] as being overexpressed in breast cancer (14 miRNAs), colon cancer (20 miRNAs) and lung cancer (33 miRNAs). We have also included a set of miRNAs that we found to be significantly overexpressed in pancreatic cancer (47 miRNAs) [8]. An additional group of seven miRNAs was reported as being overexpressed in lymphomas [10]. This group of miRNAs is encoded by a single gene (cistron miR-17-92) and expressed as a single primary transcript. Overexpression of cistron miR-17-92 was found in B-lymphomas [10] and also was shown to have strong correlation with T-lymphoma development in an animal model [11]. These datasets were selected to represent the whole spectrum of group sizes of co-expressed miRNAs that are observed in cancers: from a small set of several co-expressed miRNAs (7 miRNAs, cistron miR-17-92) to a large set (47 miRNAs, pancreatic cancer) to avoid possible bias of sample size.

### Combinatorial analysis of miRNA targets

**Figure 1:**
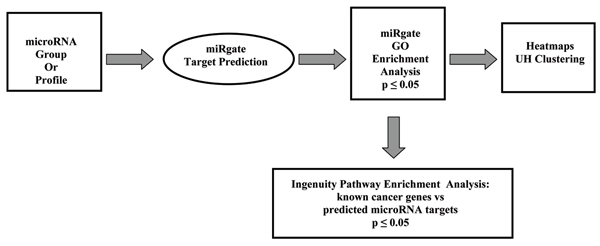
Flow Diagram of Data Analysis.

Computational analysis of predicted targets for clusters of overexpressed miRNAs was performed using novel combinatorial target prediction algorithms (miRgate 2.1 suite, Actigenics/Cepheid [16,17]). Similar to other known algorithms, a list of potential target sites (conserved between human and mouse) on 3'-UTRs of human gene-coding transcripts was determined based on a search for complimentary binding sites for the sequence of "seed" regions of 5'ends of mature miRNA positioned at 2–8 nucleotide region. These target lists were then analyzed by several statistical methods. A flow diagram of data analysis is shown on Figure 1.

### Gene Ontology enrichment analysis

As a first step, the Gene Ontology (GO) [22] enrichment analysis [23] of biological processes targeted by each of five groups of miRNAs was performed using the miRgate GO profiling algorithm that is specifically designed to take in account information about the number of miRNAs that are targeting the same genes and GO categories i.e. number of miRNA hits per GO category (miRgate 2.1 suite [16,17]). The GO categories were determined for all predicted targets of miRNAs from each of the five groups. This set of GO categories was then filtered based on significance of overrepresentation using a selected threshold for p-values of hypergeometric distribution (p ≤ 0.05) [17]. An additional filter could then be applied to select only those overrepresented GO categories which are targeted by at least several miRNAs. For our study we have generated lists of all overrepresented GO categories for each data set of over-expressed miRNAs from five types of cancer. The number of miRNAs targeting the same GO category was included in the analysis and was used as the parameter (N_m_) under investigation.

### Unsupervised hierarchical clustering of GO categories

**Figure 2:**
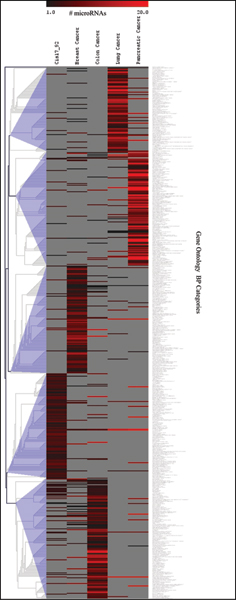
**Heatmap of unsupervised hierarchical clustering of all Gene Ontology (GO) categories obtained by enrichment analysis for 5 datasets of miRNAs**. Color gradient represents number of miRNAs targeting each category.

The resulting matrices of enriched GO categories that were affected by groups of co-expressed miRNA were further analyzed by unsupervised hierarchical clustering using information about the number of miRNAs targeted each category to find similarities and differences in patterns of affected biological functions for five types of cancer (Fig. 2). Using uncentered Pearson correlation as a distance the clusters of GO categories that were specific for each cancer were identified with a surprisingly low level of overlap.

Subsets of GO categories were generated by trimming the original matrix on the minimal number of miRNAs targeting the same category (N_m _≥ threshold) and subjected to hierarchical clustering to find categories targeted by the number of miRNAs above the threshold. Resulting clusters were compared among data sets from different cancers to determine how this trimming affects the clustering results. We observed that trimming on N_m _actually improved the separation of clusters of GO categories that are specific for each cancer.

**Figure 3:**
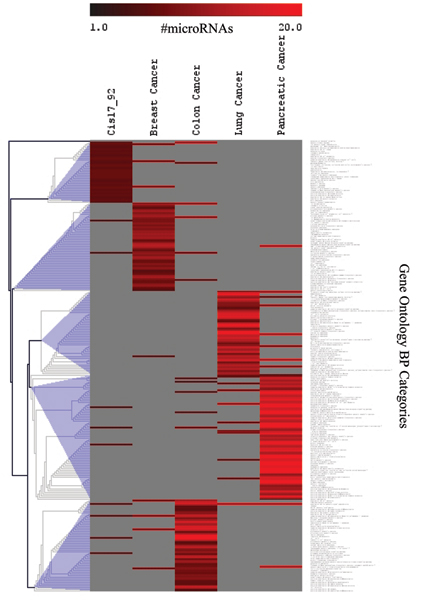
Heatmap of unsupervised hierarchical clustering of a subset of GO categories that are targeted by at least 6 miRNAs each.

**Figure 4:**
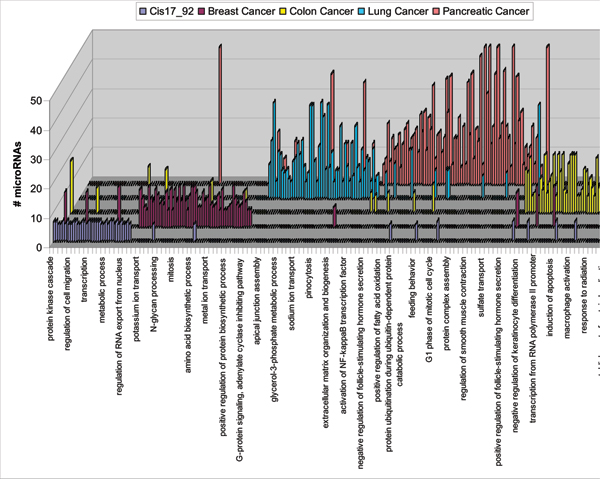
Bar graph of the same clusters of GO categories for 5 types of cancer that are presented on the Figure 3.

As an example a heat map with clustering results for N_m _≥ 6 (i.e. at least 6 miRNAs targeting each GO category) is included on Figure 3. A more detailed view of the same clusters is presented as a bar graph (Figure 4). Each bar represents a single GO category for each of five types of cancer and showing the number of miRNAs that target the same category. These clusters of GO Biological Processes contain many categories known to be affected in cancer such as general categories of proliferation, regulation of cell cycle, and transcription, as well as more specific categories such as ras-oncogene signaling pathway, chromosome segregation, and others.

### Systems Biology Analysis of predicted miRNA targets

For each set of co-expressed miRNAs we have generated 3 sets of predicted gene targets using GO ontology enrichment analysis as a statistical filter:

1. Targets that belong to GO categories targeted by at least one of the co-expressed miRNAs;

2. Targets that belong to GO categories targeted by at least 50% of the co-expressed miRNAs;

3. Targets that belong to GO categories targeted by 100% of the co-expressed miRNAs.

We started with raw lists of all targets predicted for each of the miRNAs in a range from 2175 genes (cistron mir-17-92) to 5356 genes (pancreatic cancer). Filtering raw sets of predicted targets by miRgate GO enrichment analysis algorithm provided significant reduction of target lists in a range from 2.5 fold to over 4 fold (Table 1).

**Table 1:** Number of predicted targets for 5 datasets

**miRNA Data Set**	**Number of co-expressed miRNAs**	**Total Number of Targets**	**Total number of targets from enrichment analysis (GO BP), p < 0.05**	**Number of targets from GO BP categories targeted by >50% of miRNAs**	**Number of targets from GO BP categories targeted by 100% of miRNAs**
Lymphoma (cistron miR-17-92 only)	7	2175	962	918	851
Breast Cancer	14	3462	805	720	546
Colon Cancer	20	3439	828	711	578
Lung Cancer	33	4942	700	560	192
Pancreatic Cancer	47	5356	996	841	702

Three sets of genes were generated for each of 5 groups of miRNAs and were then analyzed by Ingenuity Pathway Analysis tools to determine major biological functions, disease categories, toxicological and pathological categories, canonical signaling pathways, and drugs associated with each data set.

To select most important functional categories and pathways for analysis and to understand relevance of affected categories to the specific cancer, we generated reference sets of genes for each of five cancers by keyword search of the Ingenuity Knowledge Base. This search provided us with very conservative lists of genes that were reported to be affected in a specific cancer by multiple research groups and were then manually curated by a group of expert biologists. These 5 reference sets of genes known to be affected in lymphoma, breast cancer, colon cancer, lung cancer, and pancreatic cancer were used in the Ingenuity Pathway Analysis system to generate sets of biological functions, disease categories, and pathways known to be most affected in each of these cancers.

### Comparative analysis of biological functions and disease categories

**Figure 5:**
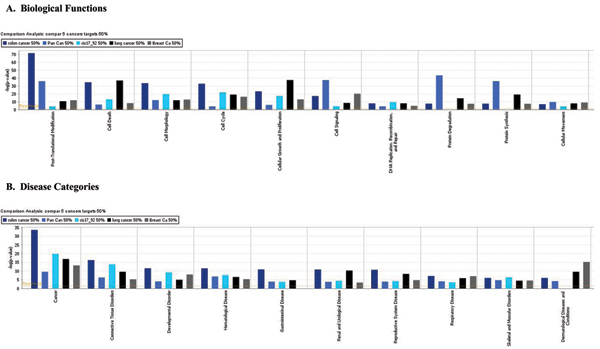
Comparison of top 10 biological function categories and disease categories enriched with miRNA target genes from 5 datasets of over expressed miRNAs.

To evaluate the specific functional categories of genes from broad GO categories that are targeted by miRNAs, we performed more detailed analysis using IPA 5.0 (Ingenuity Systems, Redwood, CA). We compared gene sets determined by GO enrichment algorithm against groups of genes known to be affected in specific cancer to determine which top ranked categories would be statistically enriched with miRNA targets. The results indicate that many top ranked biological functions and disease categories as well as toxicological categories that were tissue specific for each specific cancer were also statistically significantly overrepresented in our target lists. Top biological functions and disease related categories were compared among 5 groups of data using gene lists generated by trimming miRNA collectively targeted genes at the 50% level (Fig. 5). The top ranked disease category for all 5 datasets was Cancer (Fig. 5B) with highly significant enrichment (p ~ 10^-10 ^÷ 10^-20^). Within this top category we identified a large number of genes known as tissue specific biomarkers of each of 5 cancers. For example a list of miRNA targets for colon cancer included APC gene (adenomatosis polyposis coli) (Table 2) among other well known oncogenes. For pancreatic cancer a list of miRNA targets included both kras and p53 genes (Table 3) that are well known biomarkers of pancreatic tumors [24]. Importantly, several Ras oncogenes were experimentally validated as targets of multiple miRNAs from let-7 family [25]. Overall, in our analysis we identified 25 known cancer related genes that have been already experimentally validated as targets of miRNAs.

**Table 2:** List of predicted targets that are known genes affected in Colon Cancer

**Name**	**Description**	**Location**	**Type**	**Drugs**
AKT3	v-akt murine thymoma viral oncogene homolog 3 (protein kinase B, gamma)	Cytoplasm	kinase	enzastaurin
APC	adenomatosis polyposis coli	Nucleus	enzyme	
BAX	BCL2-associated X protein	Cytoplasm	other	
BCL2L11	BCL2-like 11 (apoptosis facilitator)	Cytoplasm	other	
BIRC5	baculoviral IAP repeat-containing 5 (survivin)	Cytoplasm	other	
BRCA1	breast cancer 1, early onset	Nucleus	transcription regulator	
CCND1	cyclin D1	Nucleus	other	
DIABLO	diablo homolog (Drosophila)	Cytoplasm	other	
EP300	E1A binding protein p300	Nucleus	transcription regulator	
ERBB3	v-erb-b2 erythroblastic leukemia viral oncogene homolog 3 (avian)	Plasma Membrane	kinase	
FASLG	Fas ligand (TNF superfamily, member 6)	Extracellular Space	cytokine	
FLT1	fms-related tyrosine kinase 1 (vascular endothelial growth factor/vascular permeability factor receptor)	Plasma Membrane	kinase	sunitinib, axitinib
FST	follistatin	Extracellular Space	other	
GSK3A	glycogen synthase kinase 3 alpha	Nucleus	kinase	
ITGB1	integrin, beta 1 (fibronectin receptor, beta polypeptide, antigen CD29 includes MDF2, MSK12)	Plasma Membrane	transmembrane receptor	
MAP2K1	mitogen-activated protein kinase kinase 1	Cytoplasm	kinase	PD 0325901
MAPK9	mitogen-activated protein kinase 9	Cytoplasm	kinase	
MAPK14	mitogen-activated protein kinase 14	Cytoplasm	kinase	SCIO-469
NFAT5	nuclear factor of activated T-cells 5, tonicity-responsive	Nucleus	transcription regulator	
NUAK1	NUAK family, SNF1-like kinase, 1	Unknown	kinase	
PARP1	poly (ADP-ribose) polymerase family, member 1	Nucleus	enzyme	INO-1001
PDCD4	programmed cell death 4 (neoplastic transformation inhibitor)	Nucleus	other	
PLK1	polo-like kinase 1 (Drosophila)	Nucleus	kinase	
PPP2R1B	protein phosphatase 2 (formerly 2A), regulatory subunit A (PR 65), beta isoform	Unknown	phosphatase	
PRKCA	protein kinase C, alpha	Cytoplasm	kinase	safingol
PRKG1	protein kinase, cGMP-dependent, type I	Cytoplasm	kinase	
SRC	v-src sarcoma (Schmidt-Ruppin A-2) viral oncogene homolog (avian)	Cytoplasm	kinase	dasatinib
TGFBR2	transforming growth factor, beta receptor II (70/80kDa)	Plasma Membrane	kinase	
YES1	v-yes-1 Yamaguchi sarcoma viral oncogene homolog 1	Cytoplasm	kinase	dasatinib

**Table 3:** List of predicted targets that are known genes affected in Pancreatic Cancer

**Name**	**Description**	**Location**	**Type**	**Drugs**
AKT1	v-akt murine thymoma viral oncogene homolog 1	Cytoplasm	kinase	enzastaurin
BCL2	B-cell CLL/lymphoma 2	Cytoplasm	other	oblimersen
CCKBR	cholecystokinin B receptor	Plasma Membrane	G-protein coupled receptor	CR 2945
CDK6	cyclin-dependent kinase 6	Nucleus	kinase	flavopiridol
CTSB	cathepsin B	Cytoplasm	peptidase	
CTSL2	cathepsin L2	Cytoplasm	peptidase	
E2F1	E2F transcription factor 1	Nucleus	transcription regulator	
FAS	Fas (TNF receptor superfamily, member 6)	Plasma Membrane	transmembrane receptor	
HGF	hepatocyte growth factor (hepapoietin A; scatter factor)	Extracellular Space	growth factor	
HMGA1	high mobility group AT-hook 1	Nucleus	transcription regulator	
IL6	interleukin 6 (interferon, beta 2)	Extracellular Space	cytokine	
KRAS	v-Ki-ras2 Kirsten rat sarcoma viral oncogene homolog	Cytoplasm	enzyme	
PLAU	plasminogen activator, urokinase	Extracellular Space	peptidase	
RHOB	ras homolog gene family, member B	Cytoplasm	enzyme	
THOC1	THO complex 1	Nucleus	other	
TOP1	topoisomerase (DNA) I	Nucleus	enzyme	elsamitrucin, T 0128, CT-2106, BN 80927, tafluposide, TAS-103, irinotecan, topotecan, 9-amino-20-camptothecin, rubitecan, gimatecan, karenitecin
TP53	tumor protein p53 (Li-Fraumeni syndrome)	Nucleus	transcription regulator	

Other top ranked categories included tissue specific diseases such as gastrointestinal systems diseases for colon cancer; reproductive systems diseases for breast cancer etc. (Fig. 5B).

The detailed information and statistics of IPA enrichment analysis for all datasets are available in Additional Files (Additional Files 1, 2, and 3). For example, the top 5 overrepresented functional categories for colon cancer included cell death, cell growth and proliferation, cell cycle, cell movement, and DNA replication and repair (Additional File 1) and we found all of these categories to be significantly overrepresented in our sets of predicted miRNA targets. Similar results were obtained for pancreatic cancer (Additional File 2).

### Comparative analysis of toxicology categories

Using IPA 5.0 we have also analyzed top ranked toxicology related gene lists for each of five reference gene lists and compared them with toxicology categories found in our miRNA target lists (Additional File 4).

We found that 8 top ranked toxicology gene lists for each cancer were statistically significantly overrepresented among miRNA targets (Additional File 4). We found it particularly interesting that several categories related to oxidative stress and hypoxia were among top ranked overrepresented categories for miRNA targeted genes (Additional File 4). These findings are in agreements with recent experimental data reporting over expression of multiple miRNAs in response to oxidative stress or hypoxia [26] and showing a functional link between hypoxia, a well-known tumor microenvironment factor, and microRNA expression.

### Comparative pathway analysis

To further evaluate the specific functions of genes from the broad GO categories that are targeted by miRNAs, we performed additional, more detailed pathway analysis (IPA 5.0, Ingenuity Systems). We compared gene sets determined by GO enrichment algorithm against known signaling pathways to determine which pathways would be statistically enriched with miRNA targets. We were also interested in determining which pathways were affected the most by multiple miRNAs from the same co-expressed group in each specific cancer.

To select pathways for analysis and to understand relevance of affected pathways to the specific cancer, we used the same reference sets of genes for each of five cancers that were obtained by keyword search of the Ingenuity Knowledge Base. These 5 reference sets of genes known to be affected in lymphomas, breast cancer, colon cancer, lung cancer, and pancreatic cancer were used in the Ingenuity Pathway Analysis system to analyze sets of pathways known to be affected in each of these cancers.

For each of the five datasets we performed analysis of all canonical signaling pathways known to be affected by each cancer to reveal pathways that are targeted by miRNAs from our datasets for each particular cancer. We used Fisher's exact test to select pathways that were statistically significantly enriched with miRNA targets (p ≤ 0.05).

The total number of targeted genes in each pathway was compared with the number of reference genes from the same pathway that are known to be affected by cancer. For each type of cancer a reference set of genes was compared with three target sets: a complete list of genes from enriched GO categories and two sets trimmed by excluding genes from those GO categories that are targeted by less than 50 percent or less than 100 percent of miRNAs in a group.

**Figure 6:**
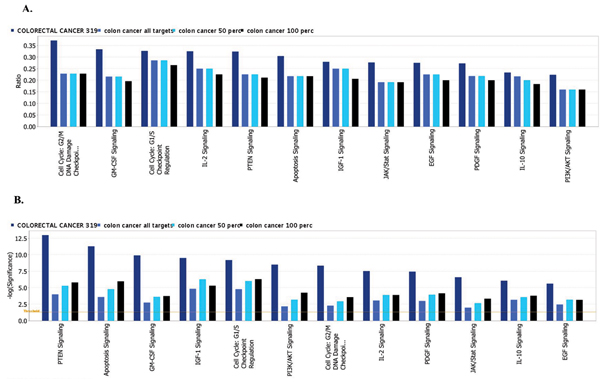
**Comparison of 12 pathways known to be affected in colon cancer and targeted by miRNAs that are over-expressed in colon cancer**. Color of the bars represents 4 data sets. Detailed description could be found in the Results section. A. Ratio of affected genes to total number of genes in the pathway. Reference gene set and three target sets are included: a complete list (all targets) and two sets trimmed by excluding genes from GO categories targeted by less than 50% or less than 100% of miRNAs in the group. B. Significance of enrichment. Threshold p < 0.05 is shown as yellow line. Bars that are above the line indicate significant enrichment of a pathway.

**Figure 7:**
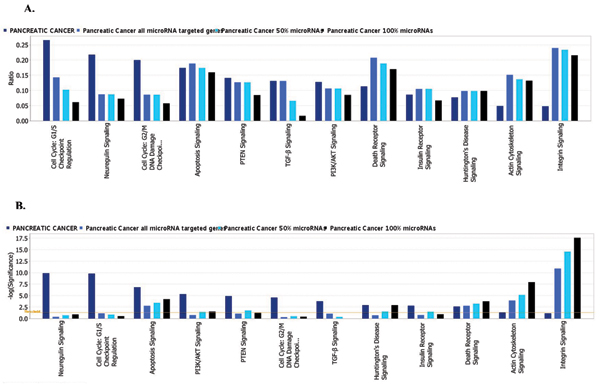
**Comparison of 12 pathways known to be affected in Pancreatic Cancer and targeted by miRNAs that are over-expressed in Pancreatic Cancer**. Color of the bars represents 4 data sets. Detailed description could be found in the Results section. A. Ratio of affected genes to total number of genes in the pathway. Reference gene set and three target sets are included: a complete list (all targets) and two sets trimmed by excluding genes from GO categories targeted by less than 50% or less than 100% of miRNAs in the group. B. Significance of enrichment. Threshold p < 0.05 is shown as yellow line. Bars that are above the line indicate significant enrichment of a pathway.

For example, Figure 6 demonstrates the results of comparison of top 12 pathways known to be affected by colon cancer and targeted by miRNAs that are over-expressed in colon cancer. Bar color represents 4 data sets: dark blue – reference gene list for colon cancer generated by Ingenuity Knowledgebase; medium blue, light blue and black – 3 gene lists that were obtained from GO enrichment analysis; a complete list (all targets) and two sets trimmed by excluding genes from GO categories targeted by less than 50% or less than 100% of miRNAs in the group. The same type of comparison of top 12 pathways for pancreatic cancer is shown on Figure 7.

Results of the pathway analysis were similar for all datasets. We found that a large fraction of pathways known to be affected in a particular cancer was also collectively targeted by co-expressed miRNAs that were experimentally detected in that cancer. However the sets of most affected pathways were specific for each type of cancer (Additional File 5).

**Figure 8:**
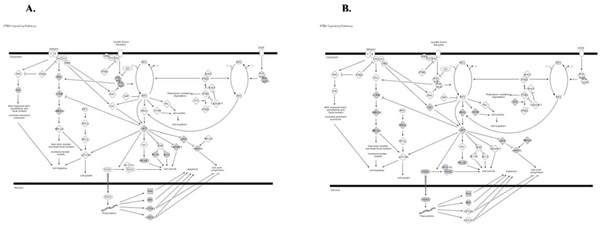
**Diagram of PTEN Signaling Pathway**. A. Genes known to be affected in Colon Cancer are shown in gray. B. Genes from significantly enriched GO categories that are targeted by at least 50% of over-expressed miRNAs from Colon Cancer dataset are shown in gray

**Figure 9:**
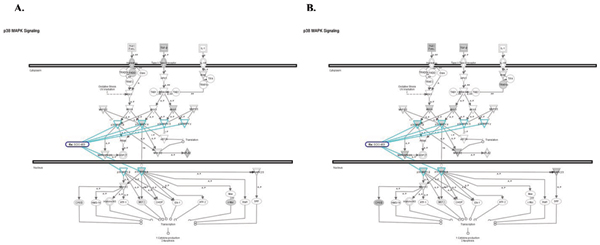
**Diagram of p38 signaling pathway**. A. Genes known to be affected in Colon Cancer are shown in gray. B. Genes from significantly enriched GO categories that are targeted by at least 50% of over-expressed miRNAs from Colon Cancer dataset are shown in gray. Drug that are known to target this signaling pathway are outlined in dark blue. Drug targets are outlined in light blue.

**Figure 10:**
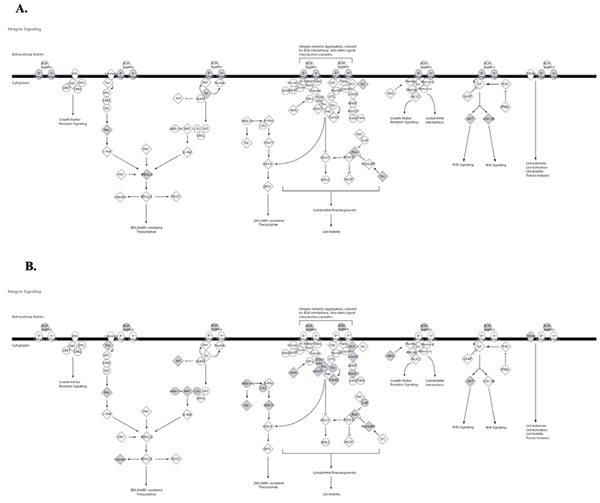
**Diagram of Integrin Signaling Pathway**. A. Genes known to be affected in Pancreatic Cancer are shown in gray. B. Genes from significantly enriched GO categories that are targeted by at least 50% of over-expressed miRNAs from Pancreatic Cancer dataset are shown in gray

**Figure 11:**
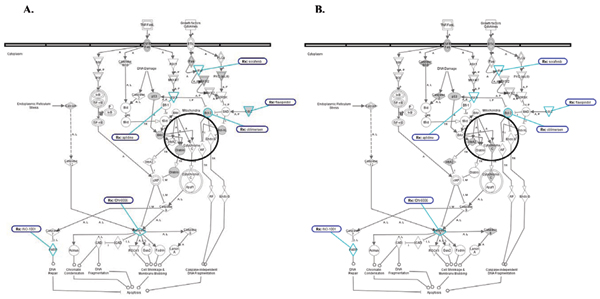
**Diagram of Apoptosis Signaling Pathway**. A. Genes known to be affected in Pancreatic Cancer are shown in gray. B. Genes from significantly enriched GO categories that are targeted by at least 50% of over-expressed miRNAs from Pancreatic Cancer dataset are shown in gray. Drug that are known to target genes from this signaling pathway are outlined in dark blue. Drug targets are outlined in light blue.

The detailed inspection of overlay diagrams revealed an interesting pattern of genes targeted by miRNAs. In the majority of inspected pathways the miRNA targets were often different from genes known to be affected by cancer and were found among genes that are directly downstream and/or upstream of the cancer related genes in the same branches of signaling cascades (Figures 8, 9, 10, 11, Additional file 6).

All overlay diagrams show target genes (highlighted in gray color) corresponding to a set of target genes from statistically enriched GO categories targeted by at least 50% of miRNAs in a group. This type of analysis was performed on all five datasets.

Two examples are shown for colon cancer (Figures 8, 9) and two – for pancreatic cancer (Figures 10, 11), with additional data presented for lymphoma in Additional File 6.

In many pathways we found that miRNAs target multiple kinases that are important mediators of signal transduction pathways and are often targeted by anticancer drugs known as kinase inhibitors (specific examples are provided in the next section). Since all miRNAs in this study were over-expressed in cancer, their overall effect would be to down-regulate many or, sometimes, all of the abnormally activated alternative signal transduction cascades in most of the pathways known to be affected by a particular cancer. Such effect would be comparable with the effect of several kinase inhibitor drugs combined.

### Anti-cancer drugs and microRNA targets

Using IPA knowledge base (Ingenuity Systems, Redwood, CA) we have analyzed known anticancer drugs and found that several drugs are targeting the same cancer related genes as the miRNAs (Tables 2, 3 and Additional Files 7, 8) including several experimentally validated microRNA targets.

One of the most common groups of drugs sharing the same targets with miRNAs was a relatively new class of kinase inhibitors that are designed to inhibit abnormally activated kinases in signal transduction pathways in cancer cells. Analysis of pathways diagrams has shown that many of these kinase inhibitors target the same kinases as do miRNAs.

For example, we found that multiple genes from p38 signaling pathway are affected in colon cancer and that kinase inhibitor drug SCIO targets all 4 izoforms of the p38 MAP kinase super-family effectively down regulating all branches of this signal transduction pathway (Figure 9A). Interestingly, three of these genes are also predicted targets of miRNAs that are co-expressed in colon cancer (Figure 9B). The p38 MAP kinase pathway plays an important function in the cellular response after infection by pathogens or inflammatory stimulation and has been also implicated in breast, colon and other types of cancer [27,28].

We found similar examples of other pathways with kinase genes that are targeted by both the anticancer drugs and miRNAs.

We have also found other types of genes that are targeted by anticancer drugs and miRNAs within the same pathways. For instance we found that apoptosis signaling pathway has many genes affected in pancreatic cancer including two critical regulators of apoptosis: Bcl-2 and Caspase-3 (Figure 11A). Bcl-2 is targeted by recently developed drug oblimesen which is an antisense synthetic oligonucleotyde-based anticancer (pro-apoptotic) drug effectively silencing Bcl-2 transcripts (Figure 11A). Capase-3 is targeted by IDN-6556, an anti-apoptotic drug (caspase inhibitor) which is indicated for hepatitis C. Both of these genes are also predicted targets of miRNAs that are over-expressed in pancreatic cancer (Fig. 11B).

Bcl-2 encodes an integral mitochondrial outer membrane protein that blocks the apoptotic death of some cells such as lymphocytes. Constitutive expression of Bcl-2 is thought to be the cause of some types of cancer [29]. Therefore down regulation of Bcl-2 by over expressed miRNAs would have pro-apoptotic anticancer effect similar to the effect of oblimesen.

Importantly, Bcl-2 is one of the few microRNA targets that were confirmed experimentally to be targeted collectively by at least two miRNAs. It has been recently shown by Cimmino et al. [29] that *miR-15a *and *miR-16-1 *expression is inversely correlated with Bcl-2 expression in CLL and that both miRNA**s **negatively regulate Bcl-2. It has also been shown that repression of Bcl-2 by these miRNAs induces apoptosis in leukemia cell lines. The authors of this study have proposed that miR-*15 *and *miR-16 *are natural antisense Bcl-2 regulators that could be used for cancer therapy of some tumors.

Paradoxically, down regulation of caspase-3 by miRNAs should have anti-apoptotic effect similar to the effect of IDN-6556 and would be beneficial for cancer cell survival. This counterintuitive mode of miRNA regulation has been recently discussed in the literature in a context of relevant experimental observations showing that miR-34a has tumor suppressor activity when ectopically expressed in NB cell lines through induction of caspase 3/7 apoptotic pathway [30]. miR-34a may have a pro-apoptotic effect, in part, through targeting the E2F3 transcription factor. Recently, it has been shown that miR-17-5p and miR-20a also act as tumor suppressors by targeting and reducing E2F1 levels [31]. Similar to miR-34a, the chromosome region with the miR-17 cluster is deleted in some human tumors. This same region, however, is amplified in diffuse large B-cell lymphoma samples [29]. Thus miRNAs may have a tumor suppressor or oncogenic effect depending upon the cell type in which they are expressed. It is also important to keep in mind that when multiple miRNAs are co-expressed within the same tumor cells a systemic regulatory impact should be considered in a context of many regulatory targets that are affected simultaneously. In case of pancreatic cancer we have found that E2F1 is a predicted microRNA target as well as caspase3. E2F1 has been also validated experimentally as a target of multiple miRNAs [30]. In this regard, it is interesting to note that similar complex regulatory responses were previously reported for the members of E2F family of transcription factors that can also have cell proliferative or pro-apoptotic effects in different cellular and regulatory contexts [[32], 33].

## Discussion

In this study we have addressed the problem of identifying major biological processes and signaling pathways that are targeted collectively by co-expressed miRNAs in cancer cells. Recently, multiple studies have reported aberrant expression profiles of miRNAome in human cancers. Several target genes were experimentally identified for some miRNAs in various tumors; however the global pattern of cellular functions and pathways affected by miRNAs in cancer remains elusive. We proposed a two step data reduction procedure and have tested it on experimentally obtained data sets of aberrantly expressed miRNAs in five human cancers.

First, we performed a statistical enrichment analysis of GO categories to find categories that are enriched with targets of co-expressed miRNAs. The miRgate GO profiling algorithm is specifically designed to take in account information about the number of miRNAs that are targeting the same genes and information about the number of miRNAs hits per GO category [17]. It allowed us to reduce a very large raw list of predicted target genes to a smaller set of target genes from significantly enriched GO categories.

Second, we have tested the idea that additional trimming of the over-represented GO categories on the total number of hits by multiple miRNAs would allow determination of those biological functions that were affected the most by miRNAs and that were more specific for each cancer. It has been shown in simulation studies [13] that the level of random noise in miRNA target predictions declined sharply even with trimming by condition of at least 3 miRNAs targeting each of the same genes. Even though we have already filtered the target list on significance of GO enrichment, we assume that using additional criteria of multiple miRNAs targeting the same categories would reduce the random predictions noise even further.

We have tested this assumption by performing unsupervised clustering analysis of GO categories using the number of miRNAs that target each category to calculate correlation-based distance and to find groups of categories that were most specifically targeted for each cancer and to determine how trimming on the number of targeting miRNAs would affect these clusters. Our results show that trimming actually improved the separation of clusters.

We have further investigated the effect of trimming in pathway analysis where only significantly overrepresented canonical pathways were selected and compared against reference gene sets known to be affected in particular cancer types. We found that for the majority of pathways the trimming on the number of miRNAs targeting the same GO category produced little or no change in the number of affected genes and at times even provided additional improvement on the significance of enrichment (Figs. 6B, 7B), most probably by eliminating unrelated genes that were diluting the target gene set.

Clustering analysis of enriched GO categories and pathways analysis of major signaling pathways have both shown that using additional criteria of multiple miRNA targeting of the same category allows narrowing down the sets of predicted targets without losing the specificity of cancer related gene lists.

Using a combinatorial target prediction algorithm we have found GO categories as well as biological functions, disease categories, toxicological categories, and regulatory pathways that are: targeted by multiple miRNAs; statistically significantly enriched with target genes; and known to be affected in specific cancers. Importantly, several well known cancer related genes such as ras, Bcl-2 and E2F1 that we identified in our analysis have been already validated in wet lab experiments and were reported by others as targets of multiple miRNAs [25,29,31]. Overall, in our analysis we identified 25 known cancer related genes that have been already experimentally validated as targets of miRNAs.

Our pathway analysis suggests that co-expressed miRNAs seem to collectively targeting a broad range of downstream signaling cascades and down regulating expression of genes in abnormally activated pathways. Such computationally inspired hypothesis could be tested experimentally by comparing microRNA expression data with mRNA expression data and protein expression data obtained from the same samples of the tumor tissue.

## Conclusion

Our global analysis of predicted miRNA targets in 5 human cancers demonstrates that co-expressed miRNAs might collectively provide systemic compensatory response to the abnormal functional and phenotypic changes in cancer cells by targeting a broad range of functional categories and abnormally activated pathways known to be affected in a particular cancer. Such systems biology based approach could provide new avenues for biological interpretation of miRNA profiling data as well as generation of experimentally testable hypotheses regarding collective regulatory functions of miRNA in cancer.

## Competing interests

The authors declare that they have no competing interests.

## Authors' contributions

YG designed and performed this study, YG and DB wrote the manuscript. TS, MG and RP provided guidance with biological interpretation, comments on the study and on the manuscript. All authors read and approved the final manuscript.

## Supplementary Material

Additional file 1 **Supplemental Figure 2 – Top ranked biological functions and disease categories targeted by miRNAs and known to be affected in Colon Cancer**. A. Top 5 Biological Functions. B. Top 5 Disease Categories Click here for file

Additional file 2 **Supplemental Figure 3 – Top ranked biological functions and disease categories targeted by miRNAs and known to be affected in Pancreatic Cancer**. A. Top 5 Biological Functions. B. Top 5 Disease Categories Click here for file

Additional file 3 Top 5 functions, disease categories and toxicology lists for 5 cancers. Click here for file

Additional file 4 **Supplemental Figure 1 – Comparison of Toxicology categories and canonical pathways targeted by miRNA for 5 types of cancer**. A. Toxicology related gene lists from top ranked Toxicology categories. B. Top ranked Canonical Pathways Click here for file

Additional file 5 **Supplemental Figure 5 – Comparison of top ranked signaling pathways targeted by microRNAs in 5 types of cancer**. A. Ratio of the number of genes targeted by miRNAs to the total number of genes in each pathway. B. Significance of overrepresentation of microRNA targets in each pathway Click here for file

Additional file 6 **Supplemental Figure 4 – TGF-b Signaling pathway**. A. Genes known to be affected in Lymphoma (shown in gray) and known anticancer drugs. B. Targets of microRNA family miR-17-92 (shown in gray) and targets of known anticancer drugs. Drug that are known to target genes from this signaling pathway are outlined in dark blue. Drug targets are outlined in light blue. Click here for file

Additional file 7 Supplemental Table 1 – Number of predicted miRNA target genes known to be associated with specific or any cancer and targeted by anti-cancer drugs. Click here for file

Additional file 8 Supplemental Tables: genes known to be affected in specific cancer and targeted by miRNAs (lymphoma, breast cancer, lung cancer). Click here for file
