# Supplementary figures and images for: Computational analysis of biological functions and pathways collectively targeted by co-expressed microRNAs in cancer
Source: BMC Bioinformatics. 2007 Nov 1;8(Suppl 7):S16. doi: 10.1186/1471-2105-8-S7-S16 (PMC2099484; doi:10.1186/1471-2105-8-S7-S16)

**Additional File 1 - Supplemental Figure 2**

**A.**

**
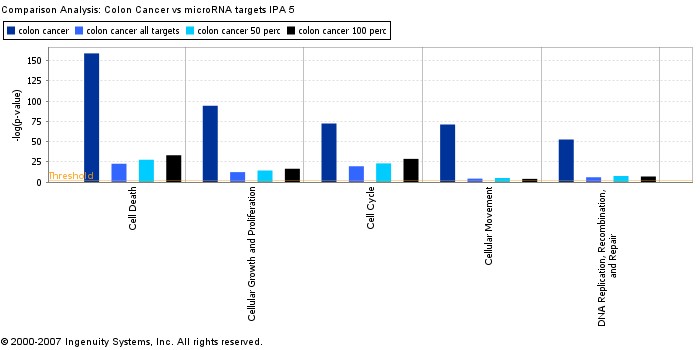
**

**B.**

**
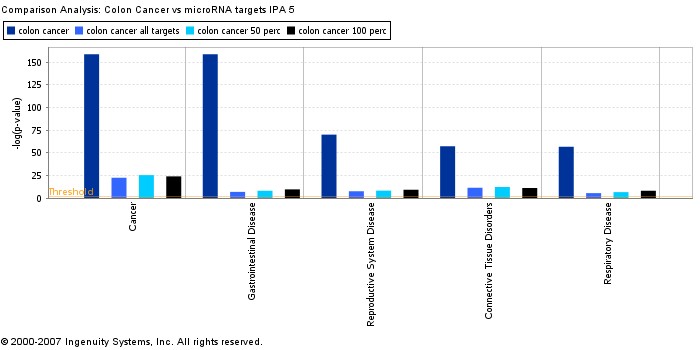
**

Supplement: Additional file 1 — Supplemental Figure 2 – Top ranked biological functions and disease categories targeted by miRNAs and known to be affected in Colon Cancer. A. Top 5 Biological Functions. B. Top 5 Disease Categories [file 1471-2105-8-S7-S16-S1.doc]

**Additional File 2 - Supplemental Figure 3**

**A.**

**
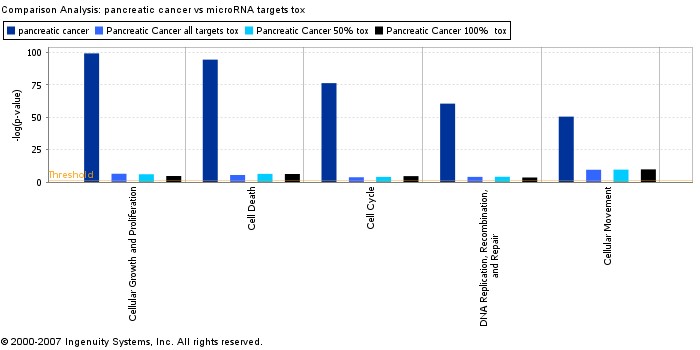
**

**B.**

**
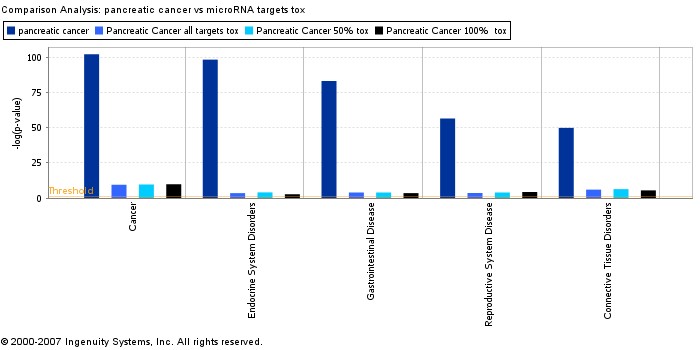
**

Supplement: Additional file 2 — Supplemental Figure 3 – Top ranked biological functions and disease categories targeted by miRNAs and known to be affected in Pancreatic Cancer. A. Top 5 Biological Functions. B. Top 5 Disease Categories [file 1471-2105-8-S7-S16-S2.doc]

**Additional File 4**

**Supplemental Figure 1**

**A. Toxicology Categories**

**
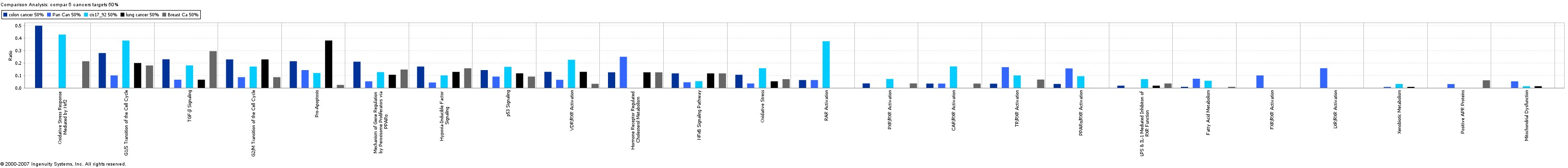
**

**B. Canonical Pathways**

**
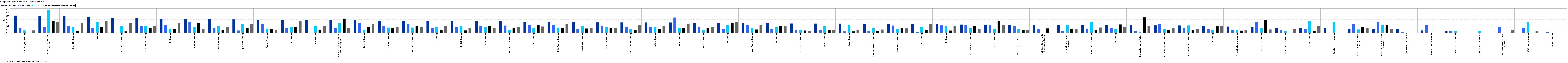
**

Supplement: Additional file 4 — Supplemental Figure 1 – Comparison of Toxicology categories and canonical pathways targeted by miRNA for 5 types of cancer. A. Toxicology related gene lists from top ranked Toxicology categories. B. Top ranked Canonical Pathways [file 1471-2105-8-S7-S16-S4.doc]

**Additional File 5 - Supplemental Figure 5**

**A.**

**
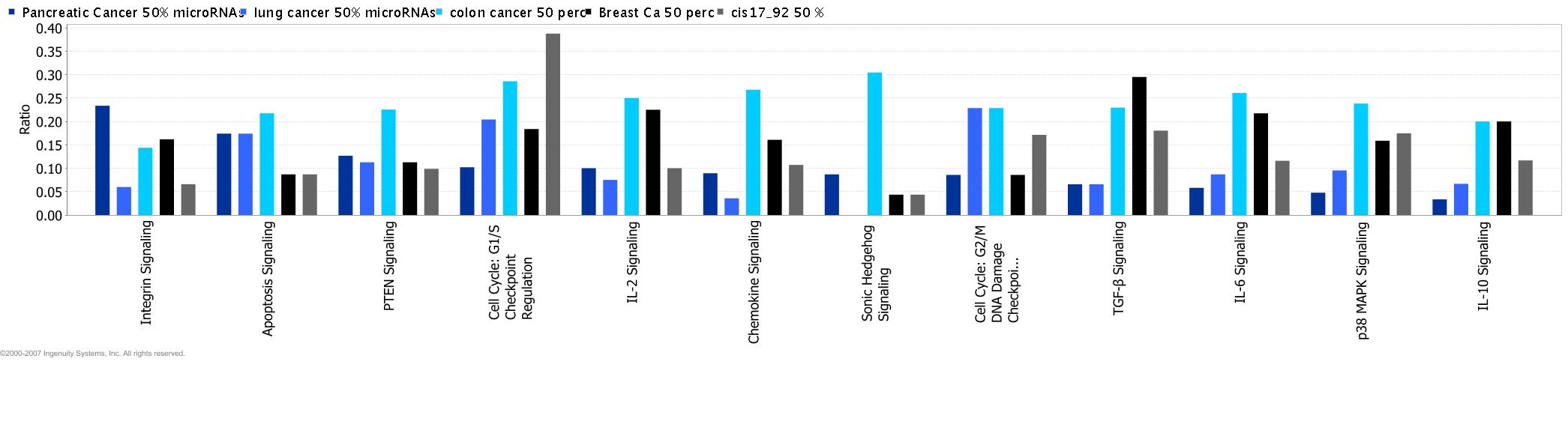
**

**B.**

**
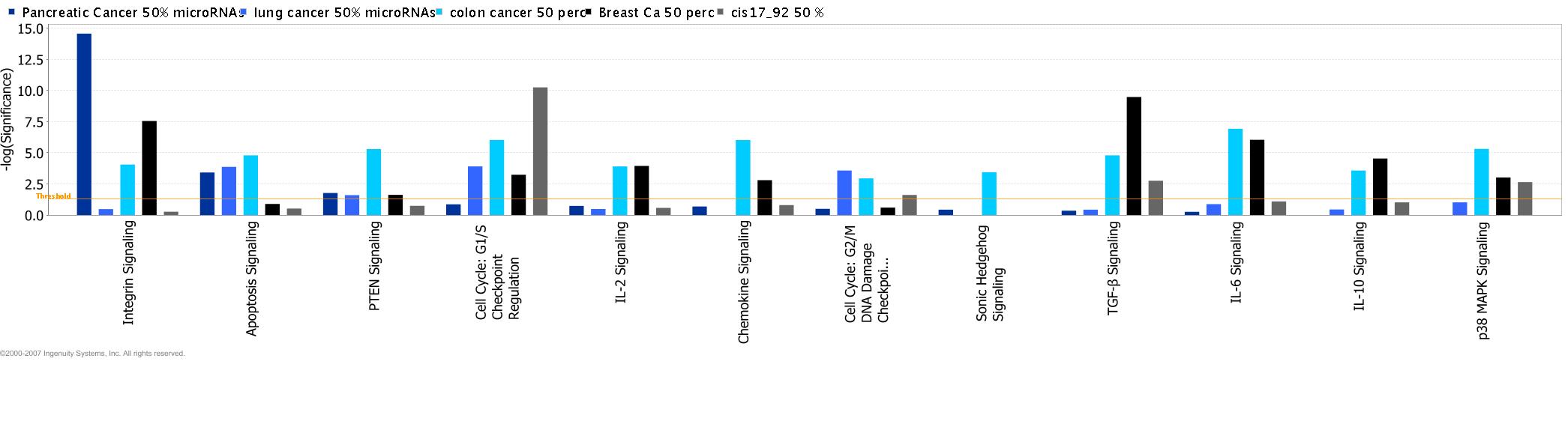
**

Supplement: Additional file 5 — Supplemental Figure 5 – Comparison of top ranked signaling pathways targeted by microRNAs in 5 types of cancer. A. Ratio of the number of genes targeted by miRNAs to the total number of genes in each pathway. B. Significance of overrepresentation of microRNA targets in each pathway [file 1471-2105-8-S7-S16-S5.doc]
