# Supplementary material for: Computational analysis of biological functions and pathways collectively targeted by co-expressed microRNAs in cancer
Source: BMC Bioinformatics. 2007 Nov 1;8(Suppl 7):S16. doi: 10.1186/1471-2105-8-S7-S16 (PMC2099484; doi:10.1186/1471-2105-8-S7-S16)
Supplement: Additional file 6 — Supplemental Figure 4 – TGF-b Signaling pathway. A. Genes known to be affected in Lymphoma (shown in gray) and known anticancer drugs. B. Targets of microRNA family miR-17-92 (shown in gray) and targets of known anticancer drugs. Drug that are known to target genes from this signaling pathway are outlined in dark blue. Drug targets are outlined in light blue. [file 1471-2105-8-S7-S16-S6.doc]

**Additional File 6 - Supplemental Figure 4**

**A. Genes known to be affected in Lymphoma B. Targets of cistron mir17-92**

**
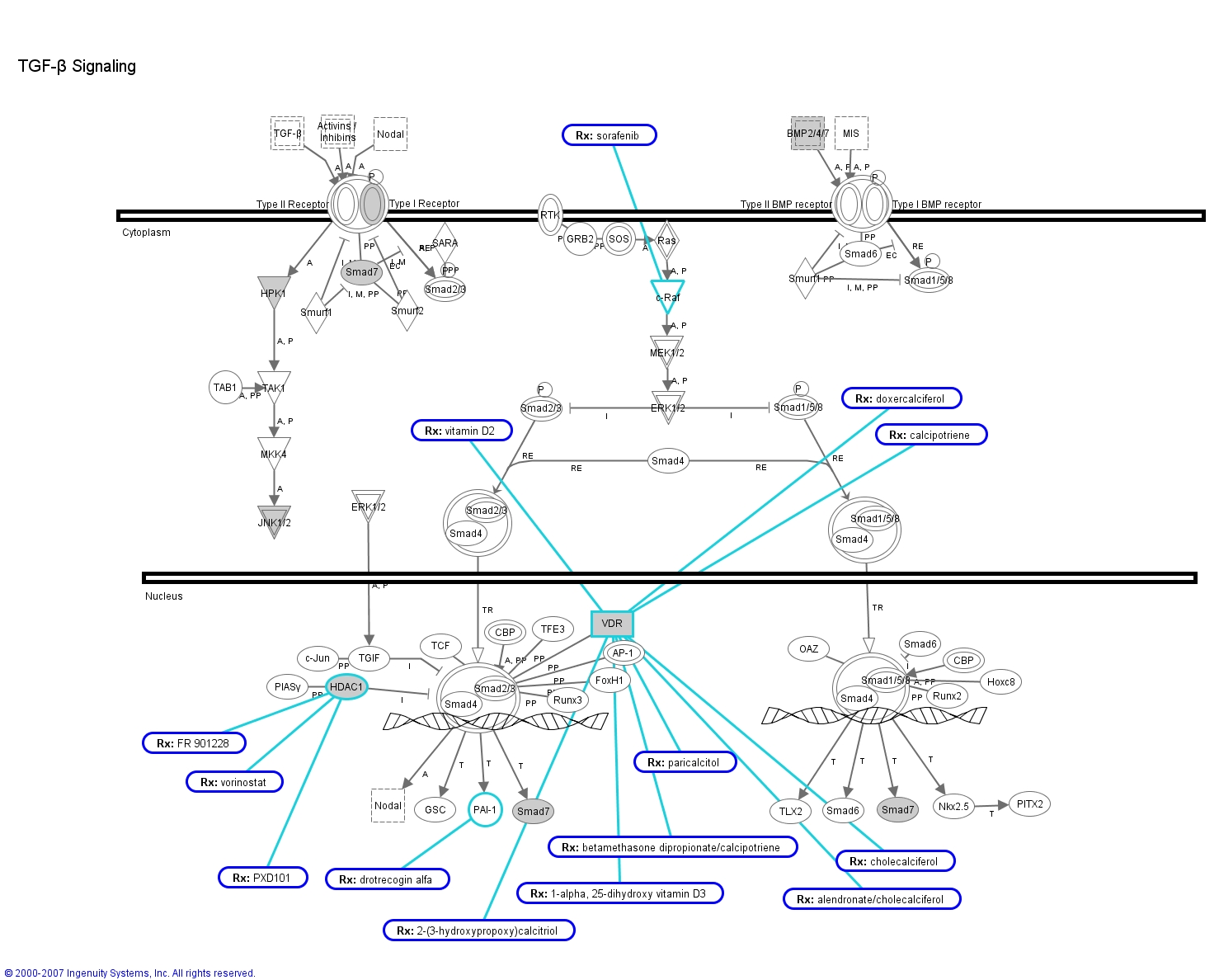

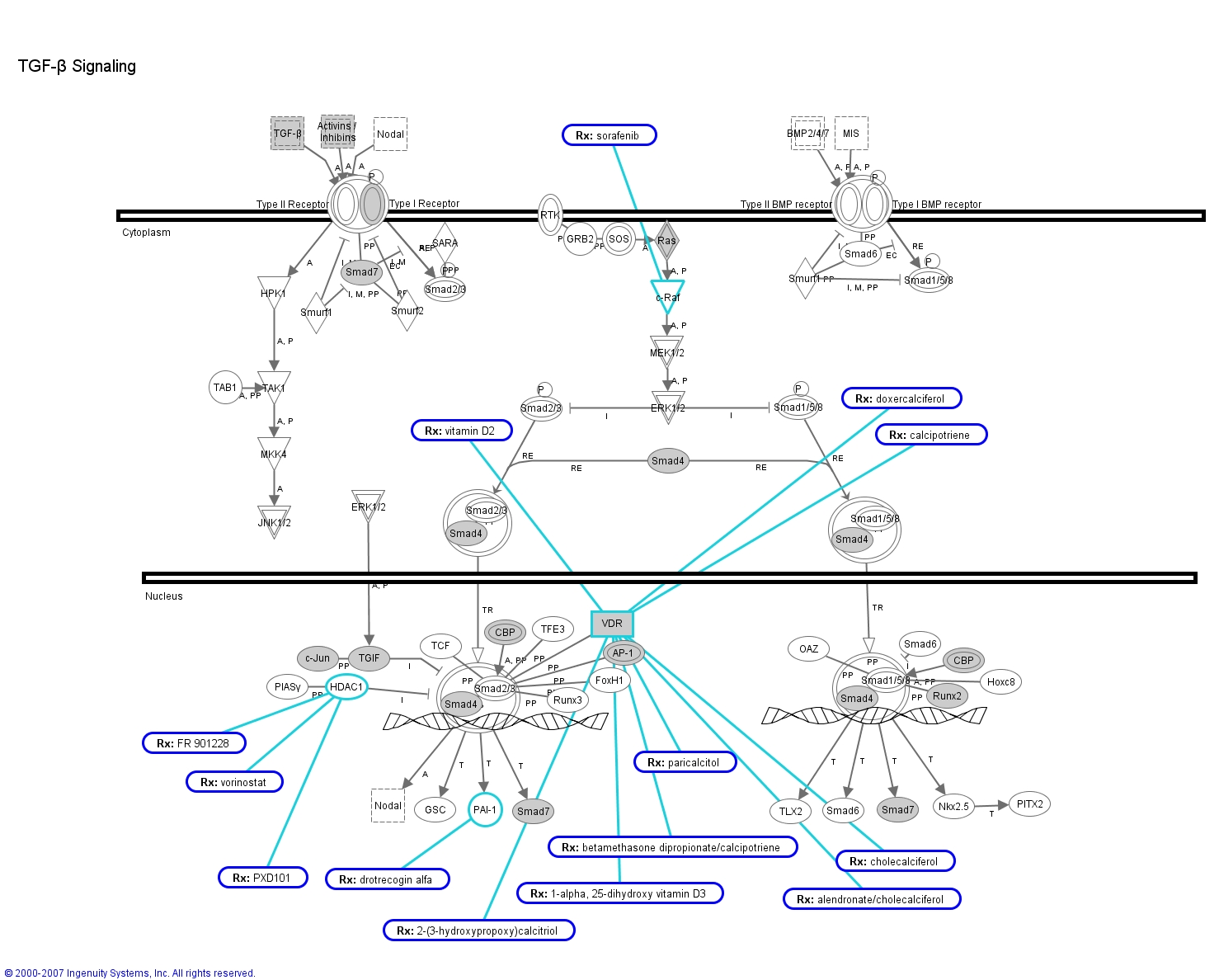
**
