# Supplementary material for: Computational analysis of biological functions and pathways collectively targeted by co-expressed microRNAs in cancer
Source: BMC Bioinformatics. 2007 Nov 1;8(Suppl 7):S16. doi: 10.1186/1471-2105-8-S7-S16 (PMC2099484; doi:10.1186/1471-2105-8-S7-S16)
Supplement: Additional file 7 — Supplemental Table 1 – Number of predicted miRNA target genes known to be associated with specific or any cancer and targeted by anti-cancer drugs. [file 1471-2105-8-S7-S16-S7.doc]

**Additional file 7**

Supplemental Table 1

| **microRNA Data Set** | **# microRNA targets associated with specific cancer** | **# microRNA targets associated with specific cancer and targeted by anti-cancer drugs** | **total # microRNA targets associated with any cancer** | **# microRNA targets associated with any cancer and targeted by anti-cancer drugs** |
| --- | --- | --- | --- | --- |
| Lymphoma (cistron miR17-92 only) | 47 | 9 | 236 | 26 |
| Breast Cancer | 67 | 10 | 179 | 22 |
| Colon Cancer | 29 | 8 | 263 | 31 |
| Lung Cancer | 37 | 9 | 184 | 24 |
| Pancreatic Cancer | 17 | 5 | 162 | 21 |
